# P-1978. Long-Term Mast Cell Activation Patterns in Systemic and Tissue-Resident Compartments Following COVID-19: Insights from a 2-Year Post-Infection Study

**DOI:** 10.1093/ofid/ofae631.2136

**Published:** 2025-01-29

**Authors:** Max Augustin, Lea Katharina Picard, Elisabeth Zenev, Melanie Ball, Isabella Rostocki, Elisabeth Pracht, Dominic Rauschning, Ute Sandaradura de Silva, Ting-Huee Lin, Sophia Petschnak, Harald Kirschner, Michael Stingl, Martin Komenda-Lett, Manuela Födinger, Christoph Wenisch, Clara Lehmann, Alexander Zoufaly

**Affiliations:** Klinik Favoriten, Vienna, Wien, Austria; University Hospital Cologne, Cologne, Nordrhein-Westfalen, Germany; Sigmund Freud University, Vienna, Wien, Austria; University Hospital Cologne, Cologne, Nordrhein-Westfalen, Germany; Sigmund Freud University, Vienna, Wien, Austria; University Hospital Cologne, Cologne, Nordrhein-Westfalen, Germany; University Hospital Cologne, Cologne, Nordrhein-Westfalen, Germany; University Hospital Cologne, Cologne, Nordrhein-Westfalen, Germany; Klinik Favoriten, Vienna, Wien, Austria; Klinik Favoriten, Vienna, Wien, Austria; Klinik Favoriten, Vienna, Wien, Austria; Facharztzentrum Votivpark, Vienna, Wien, Austria; Klinik Favoriten, Vienna, Wien, Austria; Sigmund Freud University, Vienna, Wien, Austria; Department for Infectious Diseases, Klinik Favoriten, Wiener Gesundheitsverbund, Wien, Wien, Austria; University of Cologne, Faculty of Medicine and University Hospital Cologne, Department I for Internal Medicine, Cologne, Germany, Cologne, Nordrhein-Westfalen, Germany; Department for Infectious Diseases, Klinik Favoriten, Wiener Gesundheitsverbund, Wien, Wien, Austria

## Abstract

**Background:**

The pathogenesis of Post-COVID syndrome (PCS) following mild coronavirus disease (COVID-19) caused by severe acute respiratory syndrome coronavirus type 2 (SARS-CoV-2) remains unclear. In particular, the involvement of activated mast cells (MC) in the highly angiotensin converting enzyme 2 (ACE2) expressing small bowel, the largest lymphoid organ, might be significant in PCS pathogenesis. This study aimed to elucidate the role of activated MC, residual SARS-CoV-2 spike (S) protein and zonulin (a marker for impaired intestinal integrity), in the context of PCS, examining peripheral blood (PB) and terminal ileum (TI) 2 years post-infection.

**Methods:**

In this cross-sectional, controlled study, paired PB and TI samples were obtained from 21 SARS-CoV-2 convalescents with (PCS^+^) and 11 without PCS (PCS^−^). CD117^+^CD25^+^ activated MC and residual S protein were assessed using fluorescent immunohistochemistry on sections of the the ileal tissue from the gut. Tryptase, zonulin and S protein were quantified in serum by enzyme-linked immunosorbent assay (ELISA). Flow cytometry assessed frequencies of T-cells (CD3^+^CD4^+^ (T_CD4_), CD3^+^CD8^+^ T-cells (T_CD8_)) and CCR7^±^CD27^±^ memory subsets (T_CM_: T central memory, T_EM_: T effector memory) on mononuclear cells of PB and TI.

**Results:**

Among PCS^+^ and PCS^−^, median follow up was 22 and 18 months post-infection, 81% and 73% were female, and 57% and 55% had pre-existing conditions of which arterial hypertension, chronic lung disease and diabetes mellitus were the most frequent. Compared to PCS^−^, PCS^+^ expressed significantly more MC (p< 0.0001) in ileal sections, along with elevated levels of tryptase (p=0.0196) and zonulin (p=0.024) in PB. S protein, by contrast, was undetectable in PB of PCS^+^ and PCS^−^ even 2 years post-infection. In TI however, the frequency of tissue-resident S protein-infected cells was significantly higher in PCS^+^ compared to PCS^−^ (p=0.0135). Interestingly, systemic tryptase levels correlated positively to elevated T_CM_ in TI (r=0.7407, p=0.0034).

**Conclusion:**

Elevated MC activity and S protein in the TI, along with increased tryptase and zonulin levels in PB, indicate ongoing immune activation and intestinal damage in the TI of PCS^+^. Understanding the role of tissue-resident MC in PCS pathogenesis could lead to targeted therapies.

**Disclosures:**

All Authors: No reported disclosures

